# Empathy deficit in male patients with schizophrenia and its relationships with impulsivity and premeditated violence

**DOI:** 10.3389/fpsyt.2023.1160357

**Published:** 2023-06-15

**Authors:** Muxin Gong, Lei Yao, Xiaodan Ge, Zhenru Liu, Caiyi Zhang, Yujing Yang, Nousayhah Amdanee, Chengdong Wang, Xiangrong Zhang

**Affiliations:** ^1^Department of Psychiatry, The Affiliated Xuzhou Oriental Hospital of Xuzhou Medical University, Xuzhou, Jiangsu, China; ^2^Department of Psychiatry, Xuzhou Medical University, Xuzhou, China; ^3^Department of Geriatric Psychiatry, The Affiliated Brain Hospital of Nanjing Medical University, Nanjing, Jiangsu, China

**Keywords:** schizophrenic patients, neuropsychology, violence, aggressiveness, empathy

## Abstract

**Objective:**

To explore the pattern of empathy characteristics in male patients with schizophrenia (SCH) and to examine whether empathy deficit is associated with impulsivity and premeditated violence.

**Methods:**

One hundred and fourteen male SCH patients were enrolled in this study. The demographic data of all patients were collected and the subjects were divided into two groups, namely, the violent group, including 60 cases, and the non-violent group, comprising 54 cases, according to the Modified Overt Aggression Scale (MOAS). The Chinese version of the Interpersonal Reactivity Index-C (IRI-C) was used to evaluate empathy and the Impulsive/Predicted Aggression Scales (IPAS) was employed to assess the characteristics of aggression.

**Results:**

Among the 60 patients in the violent group, 44 patients had impulsive aggression (IA) and 16 patients had premeditated aggression (PM) according to the IPAS scale. In the violent group, the scores of the four subfactors of the IRI-C, i.e., perspective taking (PT), fantasy (FS), personal distress (PD), and empathy concern (EC), were significantly lower than in the non-violent group. Stepwise logistic regression showed that PM was independent influencing factor for violent behaviors in SCH patients. Correlation analysis revealed that EC of affective empathy was positively correlated with PM but not with IA.

**Conclusion:**

SCH patients with violent behavior had more extensive empathy deficits compared with non-violent SCH patients. EC, IA and PM are independent risk factors of violence in SCH patients. Empathy concern is an important index to predict PM in male patients with SCH.

## 1. Introduction

Schizophrenia (SCH) is a spectrum of severe mental disorders which affects 1% of the population globally (1). The majority of patients experience a chronic course of the disease that might result in personality changes, social function impairment and ultimately, complete social function loss that necessitates prolonged hospitalization (2). SCH has a wide range of complex clinical manifestations which can be divided into positive symptoms (hallucinations, delusions, suspicion, anger and so on) and negative symptoms (blunted affect, emotional communication disorders, abstract thinking disorders, etc.) (3). In addition, SCH patients have aberrant mental functioning in cognition, thoughts, emotions, behaviors and other areas. SCH also disrupts social and occupational functioning and adds to the illness burden on families and the society (4, 5).

Public attention has long been drawn to the aggressive conduct of individuals with mental diseases, particularly SCH. Two meta-analyses revealed that one in 10 persons with SCH had aggressive behaviors in public, which is five times more common than in the general population (6, 7). Although instances of violence among those with schizophrenia are infrequent, it is still considered a significant issue. Unfortunately, individuals diagnosed with SCH are often unjustly stigmatized as being prone to violent behavior. It should be noted that while patients with SCH are more likely to engage in violent behaviors (8), only a fraction of violent offenders have SCH (9, 10). As a result of psychotic symptoms, SCH patients frequently exhibit aggressivity (11–13), especially during an acute episode (14, 15). Previous studies have demonstrated that patients with psychopathological conditions were more prone to engage in aggressive actions when suffering from auditory hallucinations, particularly command hallucinations (16, 17). The aggressive behaviors of hospitalized individuals typically manifest in the form of verbal aggression but can also occur as physical aggression in extreme circumstances (7, 18, 19). Violence in SCH complicates clinical treatment and management (20), raises healthcare expenses, lengthens hospital stays and exacerbates the stigma associated with the disease (21, 22).

Different focuses in multiple domains have led to a complicated definition of aggressive behavior. According the most accepted theory, aggressivity can be classified into two distinct subtypes, namely, impulsive and premeditation aggression, depending on the goal, technique and other factors (23, 24). Impulsive aggression is characterized as an emotionally charged and unrestrained type of aggression with high affective arousal and impulsivity (25) whereas premeditated assaults are planned, controlled and non-emotional actions that need foresight and planning (26, 27). While these two forms of violent conduct are independent constructs, they cannot be distinguished from one another in clinical practice and might coexist to varying degrees. Nevertheless, a wealth of research has reported that the neurobiology and neuropsychology of these two categories of aggressivity are clearly distinct (28–33). Therefore, assessing and predicting the likelihood of violent conduct in SCH patients is critical for timely management.

Empathy, the ability to comprehend the emotions of other people, is a social cognitive function that includes affiliative interpersonal communication and is linked to functional outcome in patients (34). It comprises cognitive empathy, which is defined as the identification and comprehension of another person’s emotional state and affective empathy, described as the ability to share another person’s feelings (35). While cognitive impairment is one of the primary symptoms of SCH (36, 37), research on these two forms of empathy has shown conflicting results. A previous study demonstrated that SCH patients had poor cognitive empathy, which led to difficulties in interpreting the feelings of others (38). However, a few studies revealed that emotional empathy was not reduced in SCH, showing that patients could be able to effectively experience the feelings of other people (39–41). Interestingly, violent SCH patients had lower aggressive attitudes after cognitive remediation and social cognitive training (42). Prior research indicated that violent patients exhibited cognitive deficits and higher mentalization, which might be associated with patients committing premeditated violent crimes (43–45).

To date, few researchers have investigated SCH patients who engage in aggressive behaviors. Since empathy deficiencies might be associated with aggression when violence is initiated, it is crucial to forecast the incidence of violent conduct in SCH. In this line, male SCH patients were selected in order to assess the features of violent conduct and empathy capacity as well as to investigate the link between aggressive behaviors and empathy.

## 2. Methods

### 2.1. Participants

A total of 114 male patients with SCH, aged between 18 and 55 years old, were recruited from the inpatient department of Xuzhou Oriental Hospital between September 2021 and October 2022. All the patients enrolled in the study were assessed and diagnosed with SCH by two experienced psychiatrists according to the Structured Clinical Interview for DSM-IV (SCID). The participants were classified into 60 violent schizophrenia patients (VSCH) and 54 non-violent controls (NV-SCH) according to The Modified Overt Aggression Scale (MOAS). The Chinese Interpersonal Reactivity Index (C-IRI) and the Impulsive/Premeditated Aggression Scales (IPAS) were also utilized to evaluate the patients. The patients’ general demographic information, such as age, smoking history, family history, height and weight were collected.

The exclusion criteria for all participants comprised of (1) patients with other psychotic disorders, including paranoid psychotic disorders, acute and transient psychotic disorders, schizoaffective disorders, schizotypal personality disorders, affective disorders with psychotic symptoms, mental disorders due to brain damage or physical illness and substance/drug related disorders; (2) patients suffering from psychiatric comorbidity, neurological disorders or unstable physical illnesses; (3) no alcohol consumption in the previous 30 days or drug dependence in the past 6 months.

This research adhered to the ethical principles of the World Medical Association Declaration of Helsinki (46) and was approved by the Medical Ethics Committee of Xuzhou Oriental Hospital. All the patients provided written informed consent before the start of the study.

### 2.2. Assessment instruments

#### 2.2.1. Modified overt aggression scale

All enrolled patients underwent assessment with Modified overt aggression scale (MOAS) to evaluate the frequency and severity of aggressive episodes and violence within a 1-month period (47, 48). The MOAS consists of four subscales: verbal aggression, physical aggression, self-harm and physical aggression against others (49, 50). The score of each subscale ranges from 0 to 4. A higher score indicates more severe violence, with 0 representing no violence and 4 depicting the most severe level of violence. The subscale scores are multiplied by a factor assigned to that category: 1 point for verbal aggression, 2 points for physical aggression, 3 points for self-aggression and 4 points for aggression toward others. The weighted sums of each subscale are added together to obtain the total weighted score, which ranges from 0 to 40. In this study, the violent group consisted of patients who had a MOAS total score greater or equal to 5 points (51), or an object aggression score greater than 1 point, while the non-violent control group comprised of the remaining patients (52, 53).

#### 2.2.2. Impulsive/premeditated aggression scale

The Impulsive/premeditated aggression scale (IPAS) is a reliable and valid self-report tool for assessing the occurrence of aggressive behaviors occurring over the past 6 months and categorizes aggressive conduct according to impulsive and premeditated aggressive behaviors (54, 55). The IPAS is often employed in the study of violent crimes since it can predict criminal activity (56). The scale has recently gained widespread usage globally and its Chinese version was revised in 2009 (57). The IPAS consists of 30 questions, including 8 items on impulsive aggression (IA), 12 items on premeditated aggression (PM) and 10 items on feeling in control. The items are graded on a scale of 1 to 5, whereby 1 = Strongly Disagree; 2 = Disagree; 3 = Neutral; 4 = Agree; and 5 = Strongly Agree. The proportion of positive items in the IA and PM components were determined separately using qualitative scoring. Items with scores of 5 (strongly agree), or 4 (agree) were rated as positive items, with reversed scores in the 5th and 8th items. After calculating the number of positive items, the percentage of positive items was yielded separately for both IA and PA. Participants with a higher percentage of IA positive items than PM positive items were classified as impulsive aggression (IA) subgroup. Conversely, participants with a higher percentage of PM positive items than IA positive items were classified as premeditated aggression (PM) subgroup. If the percentage of positive items was equal for both IA and PM, participants could not be classified into any subgroups. In the current study, 16 of 60 violent patients were included in the PM subgroup, while 44 of 60 violent patients were included in the IA subgroup.

#### 2.2.3. Chinese interpersonal reactivity index

The Chinese interpersonal reactivity index (IRI-C) is a test that evaluates empathy and has strong reliability and validity. Numerous investigations with Chinese participants have found that the IRI-C results are highly consistent. The IRI-C comprises 22 items which are divided into four subscales, namely, perspective taking (PT), fantasy (FS), personal distress (PD) and empathy concern (EC). While PD and EC are concentrated on the emotional empathy dimension, PT and FS are focused on the cognitive empathy aspect (58). PT measures the capacity of an individual to adopt the viewpoints of other people. FS evaluates the ability to transpose oneself into the feelings and actions of fictional characters. PD scrutinizes aversive emotional reaction that are brought on when witnessing others having negative experiences. EC examines the capacity to sympathize with people who are unfortunate (59). The PT subscale score was calculated as the average of the values for items 6, 9, 15, 19, and 22. The EC subscale score was the total of the scores for items 4, 8, 13, 18, and 21. The score for the FS subscale was the sum of items 3, 5, 10, 12, 17, and 20. The PD subscale score was determined by combining the scores of items 1, 2, 7, 11, 14, and 16. The overall scale score was calculated by adding the scores of all 22 items.

### 2.3. Statistical analysis

All statistical analyses were conducted in R software. Quantitative data were expressed as mean ± standard deviation (SD) while categorical data were described as the number of cases (*n*) and percentage (%). Independent sample *t*-test and chi-square test were utilized for demographic, self-reported violence classification, violence severity and empathy data with Bonferroni corrected comparisons. Stepwise Logistic regression analysis was used to examine the independent factors influencing aggressive behavior. A value of *p* of <0.05 was considered as statistically significant.

## 3. Results

### 3.1. Demographic characteristics

Table 1 shows the demographic data, including age, marital status, family history, total duration of illness, number of hospitalizations, weight, height and smoking history. The VSCH group had a notably higher inpatient frequency than the NV-SCH group (*p* = 0.035). Further demographic analysis between patients in the IA and PM subgroups of the VSCH group was also not statistically significantly different (*p* > 0.05).

**Table 1:** Comparison of socio-demographics and clinical variables.

	NV-SCH (*n* = 54)	VSCH		
		Total (*n* = 60)	IA (*n* = 44)	PM (*n* = 16)
Age	33.93 ± 7.89	36.23 ± 9.04	35.93 ± 9.45	37.06 ± 8.02
Marital status				
Divorced	6 (11.1)	11 (18.3)	8 (18.2)	3 (18.8)
Widowed	3 (5.6)	3 (5.0)	3 (6.8)	0 (0.0)
Unmarried	32 (59.3)	34 (56.7)	24 (54.5)	10 (62.5)
Married	13 (24.1)	12 (20.0)	9 (20.5)	3 (18.8)
Family history				
Unknown	2 (3.7)	3 (5.0)	2 (4.5)	1 (6.2)
Positive	11 (20.4)	13 (21.7)	11 (25.0)	2 (12.5)
Negative	41 (75.9)	44 (73.3)	31 (70.5)	13 (81.2)
Total disease duration	103.51 ± 76.73	120.80 ± 79.37	118.57 ± 80.40	126.94 ± 78.67
Number of hospitalizations	5.07 ± 8.87	5.60 ± 4.15^*^	5.41 ± 4.20	6.12 ± 4.11
Weight	72.31 ± 12.27	75.58 ± 11.65	76.11 ± 12.37	75.50 ± 8.96
Height	172.04 ± 5.04	172.78 ± 6.49	172.23 ± 6.10	175.00 ± 6.36
Smoking history				
No	52 (96.3)	55 (91.7)	40 (90.9)	15 (93.8)
Yes	2 (3.7)	5 (8.3)	4 (9.1)	1 (6.2)
IRI-C				
Total score	42.44 ± 13.65	33.35 ± 13.36^*^	32.20 ± 13.53*	36.50 ± 12.77
PT	11.94 ± 4.20	8.77 ± 4.37^*^	8.41 ± 4.37^*^	9.75 ± 4.36
EC	8.20 ± 4.32	6.47 ± 4.41^*^	6.41 ± 4.30	6.62 ± 4.86
FS	10.65 ± 4.60	8.28 ± 4.57^*^	7.89 ± 4.24^*^	9.38 ± 5.37
PD	11.65 ± 4.12	9.83 ± 3.59^*^	9.50 ± 3.55^*^	10.75 ± 3.68
IPAS				
Total score	68.17 ± 13.92	83.93 ± 13.64^*^	82.39 ± 13.83^*^	88.19 ± 12.53^*^
IA	17.04 ± 5.00	23.18 ± 4.45^*^	24.11 ± 4.24^*^	21.63 ± 3.56^#^^*^
PM	21.37 ± 5.43	31.60 ± 6.94^*^	29.45 ± 6.24^*^	37.50 ± 5.22^#^^*^

Data are presented as mean ± standard deviation for age, total disease duration, number of hospitalizations, weight, height, IRI-C and IPAS. Data are presented as percentage (%) for marital status, family history and smoking history. VSCH, schizophrenia patients with a history of violence; NV-SCH, schizophrenia patients without history of violence; IA, impulsive aggression; PM, premeditated aggression; IRI-C, Chinese Interpersonal Reactivity Index; IPAS, Impulsive/Premeditated Aggression Scales; PT, perspective taking; EC, empathy concern; FS, fantasy; PD, personal distress. *^#^*IA vs. PM, *p* < 0.05.

^*^ NV-SCH vs. VSCH, IA, PM, *p* < 0.05.

### 3.2. Comparison of the impulsive-premeditated aggression scale and interpersonal reactivity index-C

IRI-C intergroup comparisons revealed that the VSCH group had significantly lower IRI-C total score as well as PT, EC, FS, and PD scores compared to the NV-SCH group (*p* < 0.05). ANOVA which was conducted between the IA subgroup, PM subgroup and the NV-SCH group showed that the total IRI-C score in addition to PT, FS, and PD scores were significantly lower in the IA subgroup in contrast to the NV-SCH group (p < 0.05). No statistical differences were found in the scale ratings between the PM subgroup and NV-SCH group (*p* > 0.05). However, the IRI-C analysis did not yield statistical significance between the IA and PM groups (see Table 1).

Intergroup comparison of the IPAS demonstrated statistically significant differences between the total IPAS score as well as PM and IA component in the VSCH and NV-SCH groups (*p* < 0.05). Further analysis of variance between the IA subgroup, PM subgroup and the NV-SCH group revealed that the total IPAS score, PM and IA subscale scores were significantly higher in the IA subgroup and PM subgroup than in the NV-SCH group (*p* < 0.05). Furthermore, the IA subgroup showed higher IA subscale scores and lower PM subscale scores compared to the PM subgroup (*p* < 0.05, see Table 1).

### 3.3. Correlation analyses

In all patients, correlation analysis between the factors of empathic ability on the IRI-C and aggression characteristics of the IPAS scale revealed that PT was positively correlated with EC, FS and PD (r = 0.497, r = 0.578, r = 0.530, *p* < 0.01) while PT and FS were negatively correlated with IA (r = −0.223, r = −0.191, *p* < 0.05). Conversely, PM has a positive correlation with IA (r = 0.605, *p* < 0.01, see Table 2).

**Table 2:** Correlation analysis of all enrolled patients.

*r*	PT	EC	FS	PD	PM	IA
PT	1.000	**0.497** ^**^	**0.578** ^**^	**0.530** ^**^	−0.137	**−0.223** ^*^
EC		1.000	0.588	0.438	0.082	0.580
FS			1.000	0.518	−0.005	**−0.191** ^*^
PD				1.000	−0.730	−0.180
PM					1.000	0.605^**^
IA						1.000

IA, impulsive aggression; PM, premeditated aggression; PT, perspective taking; EC, empathy concern; FS, fantasy; PD, personal distress.

^*^ *p* < 0.05;

^**^ *p* < 0.01.

Bold values indicate statistically significant results.

In the VSCH group, correlation analysis between the factors of empathy on the IRI-C and aggression features of the IPAS scale showed that PT was positively correlated with EC, FS, and PD (r = 0.415, r = 0.523, r = 0.556, *p* < 0.01), while EC and FS were positively correlated to PM (r = 0.362, p < 0.01, r = 0.287, *p* < 0.05). EC also showed positive correlations with FS and PD (r = 0.601, r = 0.385, *p* < 0.01), while FS was positively correlated with PD (r = 0.467, *p* < 0.01, see Table 3).

**Table 3:** Correlation analysis of violent patients.

*r*	PT	EC	FS	PD	PM	IA
PT	1.000	**0.415** ^**^	**0.523** ^**^	**0.556** ^**^	0.101	−0.086
EC		1.000	**0.601** ^**^	**0.385** ^**^	**0.362** ^**^	0.177
FS			1.000	**0.467** ^**^	**0.287** ^*^	−0.035
PD				1.000	0.183	−0.004
PM					1.000	0.241
IA						1.000

VSCH, schizophrenia patients with a history of violence; IA, impulsive aggression; PM, premeditated aggression; PT, perspective taking; EC, empathy concern; FS, fantasy; PD, personal distress.

^*^ *p* < 0.05;

^**^ *p* < 0.01.

Bold values indicate statistically significant results.

In the NV-SCH group, correlation analysis between the factors of empathy capacity of the IRI-C and aggression traits on the IPAS scale indicated that PT was positively correlated to EC, FS, and PD (r = 0.528, r = 0.561, r = 0.434, *p* < 0.01). EC also showed positive correlations with FS and PD, while FS was positively correlated with PD (r = 0.530, r = 0.441, r = 0.511, *p* < 0.01). Additionally, PM showed a positive correlation with IA (r = 0.609, *p* < 0.01, see Table 4).

**Table 4:** Correlation analysis of non-violent patients.

*r*	PT	EC	FS	PD	PM	IA
PT	1.000	**0.528** ^**^	**0.561** ^**^	**0.434** ^**^	0.143	0.007
EC		1.000	**0.530** ^**^	**0.441** ^**^	0.147	0.228
FS			1.000	**0.511** ^**^	0.099	−0.095
PD				1.000	−0.003	−0.118
PM					1.000	**0.609** ^**^
IA						1.000

NV-SCH, schizophrenia patients without a history of violence; IA, impulsive aggression; PM, premeditated aggression; PT, perspective taking; EC, empathy concern; FS, fantasy; PD, personal distress.

^**^ *p* < 0.01.

Bold values indicate statistically significant results.

In the IA subgroup, correlation analysis between the factors of empathy components of the IRI-C and aggression elements of the IPAS scale demonstrated that PT was positively correlated to EC, FS and PD (r = 0.394, r = 0.522, r = 0.752, *p* < 0.01). EC also showed positive correlations with FS and PD (r = 0.614, r = 0.540, *p* < 0.01), while FS was positively correlated with PD (r = 0.613, *p* < 0.01). Additionally, EC and IA were positively correlated with PM (r = 0.321, *p* < 0.05, r = 0.466, *p* < 0.01, see Table 5).

**Table 5:** Correlation analysis of patients with IA.

*r*	PT	EC	FS	PD	PM	IA
PT	1.000	**0.394** ^**^	**0.522** ^**^	**0.752** ^**^	0.027	0.015
EC		1.000	**0.614** ^**^	**0.540** ^**^	**0.321** ^*^	0.157
FS			1.000	**0.613** ^**^	0.282	0.052
PD				1.000	0.119	0.063
PM					1.000	**0.466** ^**^
IA						1.000

IA, impulsive aggression; PM, premeditated aggression; PT, perspective taking; EC, empathy concern; FS, fantasy; PD, personal distress.

^*^ *p* < 0.05;

^**^ *p* < 0.01.

Bold values indicate statistically significant results.

In the PM subgroup, correlation analysis between the factors of empathy aspects of the IRI-C and aggression characteristics on the IPAS scale disclosed that PT and EC were positively correlated to FS (r = 0.503, r = 0.587, *p* < 0.05) and EC and IA were positively correlated with PM (r = 0.692, r = 0.746, *p* < 0.01, see Table 6).

**Table 6:** Correlation analysis of patients with PM.

*r*	PT	EC	FS	PD	PM	IA
PT	1.000	0.474	**0.503** ^*^	−0.025	0.067	−0.208
EC		1.000	**0.587** ^*^	0.013	**0.692** ^**^	0.305
FS			1.000	0.113	0.179	−0.066
PD				1.000	0.132	0.029
PM					1.000	**0.746** ^**^
IA						1.000

IA, impulsive aggression; PM, premeditated aggression; PT, perspective taking; EC, empathy concern; FS, fantasy; PD, personal distress.

^*^ *p* < 0.05;

^**^ *p* < 0.01.

Bold values indicate statistically significant results.

### 3.4. Logistic regression analysis

A one-way logistic regression analysis was performed on the demographic data and scale scores of all patients in order to analyze the factors influencing the occurrence of violent behavior in patients with SCH and we found that PT (OR = 0.840, 95% CI 0.762–0.925, *p* < 0.001), EC (OR = 0.913, 95% CI 0.837–0.995, *p* = 0.039), FS (OR = 0.892, 95% CI 0.819–0.972, *p* = 0.009), PD (OR = 0.883, 95% CI 0.798–0.977, *p* = 0.016), PM (OR = 1.283, 95% CI 1.164–1.387, *p* < 0.001), and IA (OR = 1.311, 95% CI 1.141–1.373, *p* < 0.001) were statistically significant (see Table 7). The factors with statistical significance in univariate logistic regression analysis were included in multivariate logistic regression analysis. The analysis revealed that the affective empathy, EC (OR = 0.776, 95% CI 0.669–0.901, *p* = 0.001), and the aggressive trait, PM (OR = 1.260, 95% CI 1.136–1.397, *p* < 0.001) and IA (OR = 1.244, 95% CI 1.072–1.443, *p* = 0.004), were significant predictors of violent behavior in patients with SCH.

**Table 7:** Univariate analysis of violent patients.

Variables	*OR*	*95% CI*	*P*
Age	1.033	0.988–1.080	0.152
Marital status	1.111	0.741–1.666	0.612
Family history	1.081	0.438–2.668	0.865
Total disease duration	1.003	0.998–1.008	0.241
Number of hospitalizations	1.012	0.956–1.072	0.681
Weight	1.024	0.992–1.058	0.149
Height	1.022	0.959–1.090	0.495
Smoking history	2.364	0.439–12.722	0.316
PT	0.840	0.762–0.925	**<0.001** ^*^
EC	0.913	0.837-0.995	**0.039** ^*^
FS	0.892	0.819-0.972	**0.009** ^*^
PD	0.883	0.798-0.977	**0.016** ^*^
PM	1.283	1.164-1.387	**<0.001** ^*^
IA	1.311	1.141-1.373	**<0.001** ^*^

Significant univariate variables (*p* < 0.05) were included in a multivariate regression analysis and stepwise logistic regression was used for variable screening. The affective empathy, EC (OR = 0.776, 95% CI 0.669–0.901, *p* = 0.001), and the aggressive trait, PM (OR = 1.260, 95% CI 1.136–1.397, *p* < 0.001) and IA (OR = 1.244, 95% CI 1.072–1.443, *p* = 0.004), were significant predictors of violent behavior in patients with SCH.

^*^ *p* < 0.05.

Bold values indicate statistically significant results.

## 4. Discussion

This study examined the empathy deficit in male violent schizophrenic patients and its correlation with impulsive and premeditated violence. Empathy abilities were significantly impaired in VSCH patients compared to NV-SCH patients, but no significant difference was observed in IA and PM subgroups. The results of the correlation analysis examining the relationship between empathy, impulsivity, and premeditated violence indicate a positive correlation between affective empathy and premeditated violence among VSCH, IA, and PM patients. Specifically, the analysis reveals that EC in affective empathy is positively associated with premeditated violence. Regression analysis showed that EC, PM and IA significantly predicted violent behaviors in male patients with schizophrenia. The results align with earlier theories that patients with VSCH exhibit broader empathy impairment in contrast to those with NV-SCH. Additionally, it suggests that premeditated violence is linked with affective empathy.

### 4.1. Empathy deficits and violent behavior in schizophrenia

In the present study, there were significant differences in empathy among VSCH patients compared to NV-SCH patients. In line with prior studies (44, 60–62), the VSCH patients in the present paper had considerably more impaired empathy, particularly affective empathy and cognitive empathy. Research indicates that violent patients face challenges with empathic reasoning. Additionally, exploratory regression analyses suggest that violent behavior correlates with impaired empathy (43). It was previously shown that the ability to identify others’ emotions and facial expressions were significantly impaired in SCH patients (63, 64), which is thought to play a significant role in social dysfunction. In a study involving patients with chronic SCH, PD in the IRI was identified as a risk factor of suicide which is a serious and violent act against oneself (65). These results suggest that empathy deficits are core characteristics of SCH patients with violent behaviors. Empathy is crucial in comprehending the emotions and sentiments of others. However, impaired empathy can result in a prejudiced view of others’ motives and the belief that they are concealing their true intentions. This can then trigger a sense of victimization and promote aggressive conduct in patients.

In the investigation of the neural basis of empathy, a meta-analysis reported that independent of specific task or stimulus type, there was a sustained activation of the dorsal anterior cingulate cortex-anterior midcingulate cortex-supplementary motor area and bilateral insula, forming a core empathy network (66). One study found that structural alterations and disturbed resting-state functional connectivity in the core empathy network might serve as the neural foundation of social cognitive deficits in individuals with early-onset SCH (67). Another research demonstrated that empathy deficits were associated with lower activation of the amygdala (68). The alterations in these brain regions might underlie the mechanisms for empathy deficits in VSCH patients.

### 4.2. Empathy deficits and IPAS in VSCH

The study discovered that VSCH patients with the IA subgroup experience more severe empathy deficits. Comparatively, the IA subgroup showed lower scores in the PT, FS, and PD dimensions of the IRI-C scale than other NV-SCH patients. Previous studies suggest that impulsive aggression is correlated with high levels of guilt, hostility, neuroticism, and trait anger (56, 69, 70), but few studies have delved into the empathy of IA subgroups. The frustration-aggression hypothesis (71) suggests that negative emotional states stemming from frustration or social pressure could lead to anger and an increase in impulsive aggression (72). In addition, a lack of empathic ability may exacerbate a patient’s anger and contribute to impulsive aggression.

### 4.3. Correlations between IRI-C factors and IA of IPAS in all SCH patients

This study reveals a correlation between higher IA scores and lower cognitive empathy scores (PT and FS) in all SCH patients. Cognitive empathy, the ability to comprehend the emotions of others (73, 74), plays a crucial role in social cognition and significantly impacts the social function of patients (75). Earlier research suggests that cognitive empathy can explain changes in social functioning, such as community functioning, in SCH patients (76). This indicates that patients with SCH who exhibit less impairment of cognitive and emotional empathy tend to understand and care for others while showing less impulsive and aggressive behaviors. However, ignoring others’ emotions can lead to impulsive aggression. The study also found a significant association between PT and FS, PD, and EC in all SCH patients, indicating consistent cognitive and affective empathy deficits.

### 4.4. Correlations between IRI-C factors and PM of IPAS in VSCH

This study yielded results regarding the correlation analysis conducted on IRI-C and IPAS scales in VSCH patients. Specifically, the EC dimension in affective empathy showed a positive correlation with PM scores in a sample population of VSCH patients, as well as IA and PM patients. Prior researches on schizophrenia patients have demonstrated impaired emotional empathy (40, 77). Moreover, literatures have shown a relationship between premeditated aggression and psychopathic personality traits, with premeditated aggression being more associated with the latter than impulsive aggression (78, 79). Individuals demonstrating lack of empathy, remorse, and guilt, alongside manipulative, callous, and grandiose behaviors (80, 81) may exhibit premeditative aggressive behavior. Experiencing affective empathy deficits can result in patients being unable to empathetically respond to the situations and experiences of others, which can increase the likelihood of engaging in premeditated aggression.

### 4.5. Independent risk factors for violence in SCH

Our study indicates that premeditated aggression scores are linked with aggressive behavior in SCH patients, establishing premeditated aggression trait in these patients as a significant predictor for violent aggression. Premeditated aggression is a learned behavior that is reinforced by receiving rewards. Extensive research evidences suggest that premeditated violence is associated with psychopathic traits (82–84). Antisocial personality disorder (ASPD) is a psychological condition often distinguished by the presence of psychopathy attributes. ASPD is commonly identified in schizophrenic patients with a previous history of violence (85). It is imperative to conduct more research to determine whether the predictive value of premeditated aggression persists after considering the effects of psychopathy on violent behavior among schizophrenia patients. This will aid in identifying suitable measures for early detection, prevention goals and approaches, and reducing the stigma accompanying this disorder.

In addition, patients in the SCH group with higher IA scores were more likely to commit violent acts. This type of aggression is largely linked to negative emotions and psychiatric symptoms, and has been shown to contribute to higher levels of guilt, hostility, and trait anger (79, 86). Negative emotional states resulting from stress and stimulation can lead to anger, which in turn increases the likelihood of impulsive aggression. This behavior is strongly associated with increased volume of the left putamen and decreased volume of the right middle temporal gyrus, superior temporal gyrus, and insula, structures involved in processing environmental stimuli and impacting aggression threshold (87–89). Given these findings, it is important to prioritize attention to male SCH patients displaying impulsive aggression, as their violent behavior is more easily triggered and poses a risk to individuals and property.

Male schizophrenia patients with impaired EC were found to be at higher risk of displaying aggressive behavior. EC refers to empathy for less fortunate others and is associated with improved emotion recognition (90). Empathy interventions have been shown to improve empathy in offenders, with those with more empathy impairment having higher rates of crime (91). These findings suggest the importance of early intervention strategies to improve cognitive empathy and prevent violent incidents.

### 4.6. Limitations

This study has several limitations that should be acknowledged. The small sample size of the current study makes the results potentially less reliable. The lack of differences in demographic and clinical characteristics in the present study is inconsistent with the results of previous investigations and might be attributed to the small sample size. In the future, the sample size should be further expanded to reduce errors. In this study, there were no healthy controls and thus, we cannot provide further evidence that SCH patients had a deficit in empathy. In addition, the study was designed as cross-sectional research, which makes it impossible to confirm whether there is a direct causal relationship between violent behavior and empathy in SCH patients. Moreover, although IRI-C is widely used to assess empathy, it is a self-report measurement. Multidimensional assessment methods are needed for future studies. Furthermore, we did not take female patients, medication status and education level into account and these factors might affect the findings of this study.

## 5. Conclusion

In summary, the VSCH patients had a more extensive empathy deficits compared to NV-SCH patients. EC, IA and PM were independent influences of violent behavior in male SCH patients. Further analysis revealed that deficits in the empathic capacity in male SCH patients were closely and positively correlated with PM characteristics, while they were not significantly correlated with IA aggression characteristics. These findings could be used to predict the occurrence of premeditated aggression in male SCH patients via empathy assessments. In addition, targeted interventions for empathic competence would be beneficial in reducing the incidence of premeditated aggression in SCH patients.

## Data availability statement

The raw data supporting the conclusions of this article will be made available by the authors, without undue reservation.

## Ethics statement

The studies involving human participants were reviewed and approved by Xuzhou Oriental Hospital Ethics Committee. The patients/participants provided their written informed consent to participate in this study. Written informed consent was obtained from the individual(s) for the publication of any potentially identifiable images or data included in this article.

## Author contributions

MG, LY, XG, ZL, CZ, YY, NA, CW, and XZ contributed equally to each stage of the study design, data collection, analysis, and write-up. All authors contributed to the article and approved the submitted version.

## Funding

This project was funded by National Natural Science Foundation of China (General Program) (81971255), Jiangsu Provincial Department of Science and Technology Social Development Project (BE22019610), Jiangsu Province Frontier Leading Technology Basic Research Project “Robot Emotion Recognition and Interaction Technology Foundation” (BK20192004D), Key Project of Medical Talents in Jiangsu Province (ZDRCA2016075), and Xuzhou Science and Technology Project (KC21181).

## Conflict of interest

The authors declare that this research was conducted in the absence of any commercial or financial relationships that could be construed as a potential conflict of interest.

## Publisher’s note

All claims expressed in this article are solely those of the authors and do not necessarily represent those of their affiliated organizations, or those of the publisher, the editors and the reviewers. Any product that may be evaluated in this article, or claim that may be made by its manufacturer, is not guaranteed or endorsed by the publisher.
